# Hyperbaric oxygen therapy for post-partum bell's palsy associate with anti-phospholipid syndrome: a case report, literature review, and mechanistic insights

**DOI:** 10.3389/fresc.2026.1665217

**Published:** 2026-03-04

**Authors:** Vicktoria Elkarif, Eli Kravchik, Liat Morgen, Shai Efrati

**Affiliations:** 1 Sagol Center for Hyperbaric Medicine and Research, Shamir (Assaf Harofeh) Medical Center, Zerifin, Israel; 2 Physical Therapy Department, Shamir Medical Center, Zerifin, Israel; 3 Independent Researcher and One Welfare Consultant, Qidron, Israel; 4 Faculty of Medical and Health Sciences, Gray School of Medicine, Tel Aviv University, Tel Aviv, Israel; 5 Sagol School of Neuroscience, Tel-Aviv University, Tel-Aviv, Israel

**Keywords:** anti phospholipid syndrome, Bell's palsy, case report, hyperbaric oxygen therapy, post partum

## Abstract

**Background:**

Bell's palsy, the most common cause of acute facial paralysis, can occur more frequently during pregnancy and postpartum due to physiological changes such as fluid retention, hormonal shifts, and immune modulation. Antiphospholipid syndrome (APS), an autoimmune disorder characterized by thrombosis and pregnancy related complications, further increases the risk of microvascular ischemia affecting cranial nerves.

**Case presentation:**

We report the case of a 37-year-old woman with longstanding APS who developed postpartum Bell's palsy unresponsive to corticosteroid therapy. Despite receiving 60 mg of prednisone, no clinical improvement was noted. Hyperbaric oxygen therapy (HBOT) was initiated, prescribed for 20 sessions at 2 absolute atmospheres, 5 days per week. Remarkable improvement was observed after the first session, with complete symptom resolution by the twelfth session. Additionally, anti-dsDNA antibodies, previously positive during pregnancy, became negative following HBOT.

**Conclusion:**

HBOT may represent a valuable adjunctive treatment for postpartum Bell's palsy in high-risk populations such as those with APS, offering combined benefits of enhanced oxygenation, inflammation control, and vascular repair. Further prospective studies are warranted to validate these findings and define standardized treatment protocols.

## Introduction

Bell's palsy is an acute, idiopathic, and unilateral peripheral facial nerve paralysis characterized by sudden onset of muscle weakness on one side of the face, with an annual incidence of 11–40 per 100,000 person per years (1). While its pathophysiology remains uncertain, proposed mechanisms include viral reactivation, autoimmune processes, and ischemia of the facial nerve (2, 3). Approximately 70% of cases recover fully (2), while the remaining patients experience varying degrees of residual weakness, synkinesis, and aesthetic or functional deficits (4).

Pregnant and postpartum women, exhibit a higher incidence of Bell's palsy, particularly in the third trimester and early puerperium (5), with recovery rates significantly lower than in non-pregnant women of similar age (52% vs. 77%–88%) (5). Physiological changes during pregnancy including fluid retention, hypercoagulability, hormonal shifts, and immune modulation, can contribute to nerve compression, perineural edema, or thrombosis of the vasa nervorum, compromising facial nerve perfusion (6, 7). Associations between Bell's palsy and preeclampsia (8) reinforce vascular ischemia as key pathophysiologic mechanisms.

Women with antiphospholipid syndrome (APS), an autoimmune condition linked with systemic lupus erythematosus, are at particularly high risk (9). APS is characterized by thrombotic events and pregnancy complications such as preeclampsia, recurrent miscarriage, and placental insufficiency. In APS, vasa nervorum thrombosis, endothelial dysfunction, and inflammatory cascades can contribute to facial nerve ischemia (10).

Corticosteroids remain the first-line treatment for Bell's palsy (11), although their use during pregnancy and postpartum is sometimes limited due to safety concerns (12). Hyperbaric oxygen therapy (HBOT) has emerged as a promising adjunct facilitating nerve regeneration by enhancing tissue oxygenation, reducing inflammation, and promoting neurogenesis (13). Animal studies show that HBOT, especially when combined with corticosteroids, reduces axonal degeneration and vascular obstruction while increasing axonal diameter, indicating synergistic effects (14). Clinical studies have similarly suggested improved recovery rates with HBOT compared to corticosteroid therapy alone, although some studies are limited by methodological constrain (15). Recent case series have demonstrated the efficacy of combined HBOT and corticosteroids for Bell's palsy in non-pregnant and otherwise healthy individuals (16). However, the current report is unique in addressing the intersection of pregnancy, APS, and HBOT. The presence of APS introduces additional thrombotic and immunological risks, which may render standard management less effective; thus, several case reports and small clinical series have shown mild clinical improvement with HBOT in patients with APS (17, 18).

This case report underscores the need for tailored therapeutic strategies in complex patients, particularly in the context of postpartum Bell's palsy associated with APS, where pregnancy-related physiological changes, nerve ischemia, and immune dysregulation likely contribute to pathophysiology. HBOT was employed to improve nerve oxygenation, reduce inflammation and edema, relieve nerve compression, and promote recovery with complete resolution of symptoms observed, supporting HBOT as a valuable adjunct in managing facial nerve palsy associated with pregnancy and APS. The findings highlight the potential benefit of HBOT in refractory cases and encourage further investigation into its role in autoimmune-mediated and pregnancy-associated Bell's palsy.

## Case presentation

A 37-year-old Caucasian woman with a longstanding diagnosis of double-positive APS presented to our clinic with Bell's palsy following the delivery of her second child. A month before delivery, she developed pulsatile tinnitus in the right ear. On the day of delivery, she presented to the emergency department (ED) with blurry vision, headache, and mild numbness of the right side of her lip. While in the ED, labor commenced. Twenty-four hours after delivery, she developed progressive weakness and paralysis of the right side of her face. A brain CT ruled out a stroke, while MRI revealed isolated enhancement of the right seventh cranial nerve, consistent with Bell's palsy.

The patient had a history of APS for a decade, with prior plantar vein thrombosis treated with aspirin 100 mg daily. During her first pregnancy, she received enoxaparin 40 mg prophylactically, which was complicated by placental abruption and preterm delivery at 35 weeks, followed by bilateral Portal vein thrombosis. In her second pregnancy, she received enoxaparin 80 mg and aspirin 100 mg, delivering a healthy child at 37 weeks. Over the years, she tested positive for anti-ANA antibodies but did not meet criteria for systemic lupus erythematosus. During her second pregnancy, anti-dsDNA antibodies emerged, and she became triple-positive for APS with the addition of lupus anticoagulant. She was evaluated by a multidisciplinary team including neurology, ENT, ophthalmology, rheumatology, and hematology, who all agreed on Bell's palsy as the diagnosis, excluding other thrombotic events.

**Figure 1:**
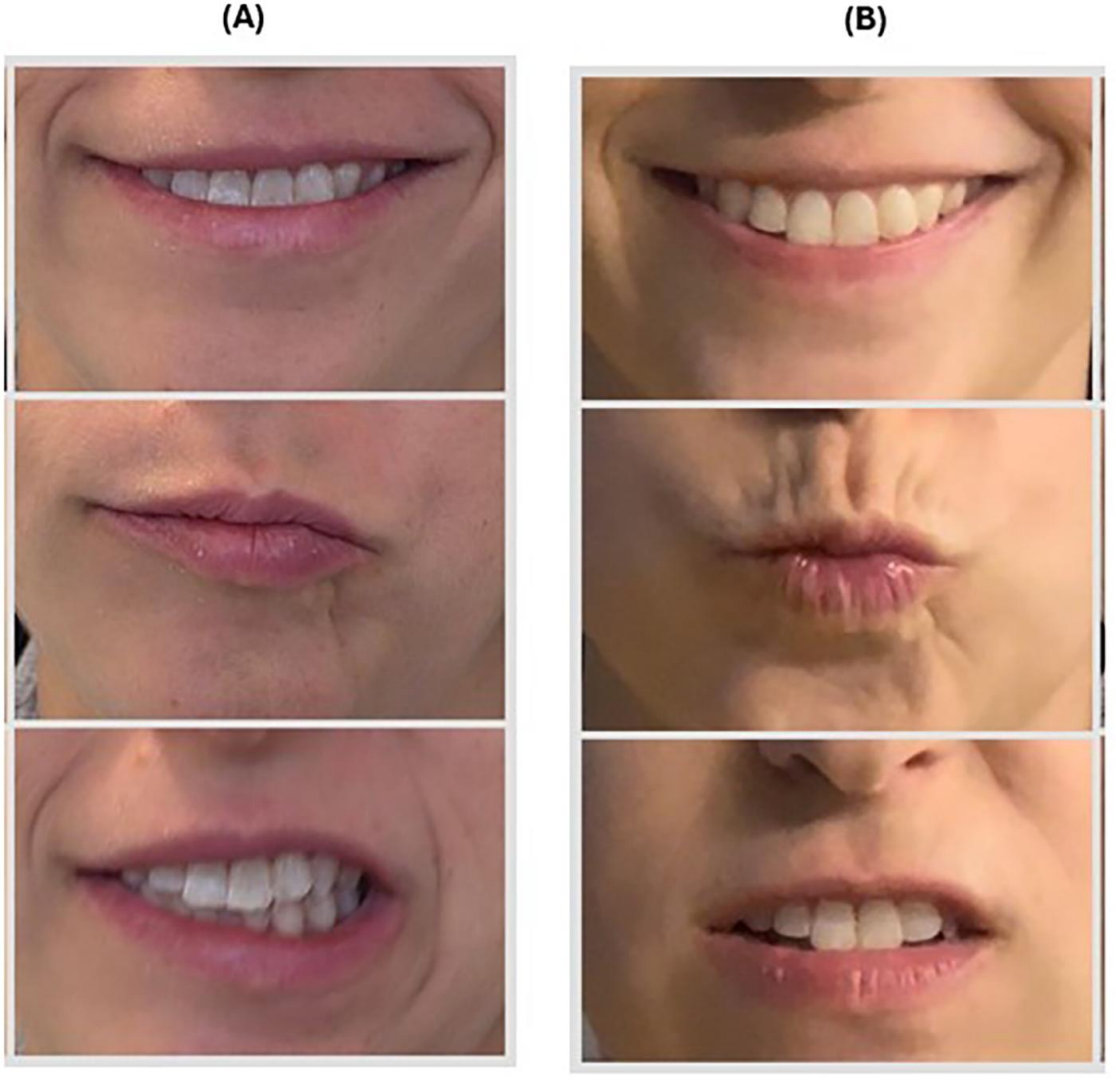
Facial function pre hyperbaric therapy treatment **(A)** and post 10 hyperbaric therapy treatment **(B).**

**Figure 2:**
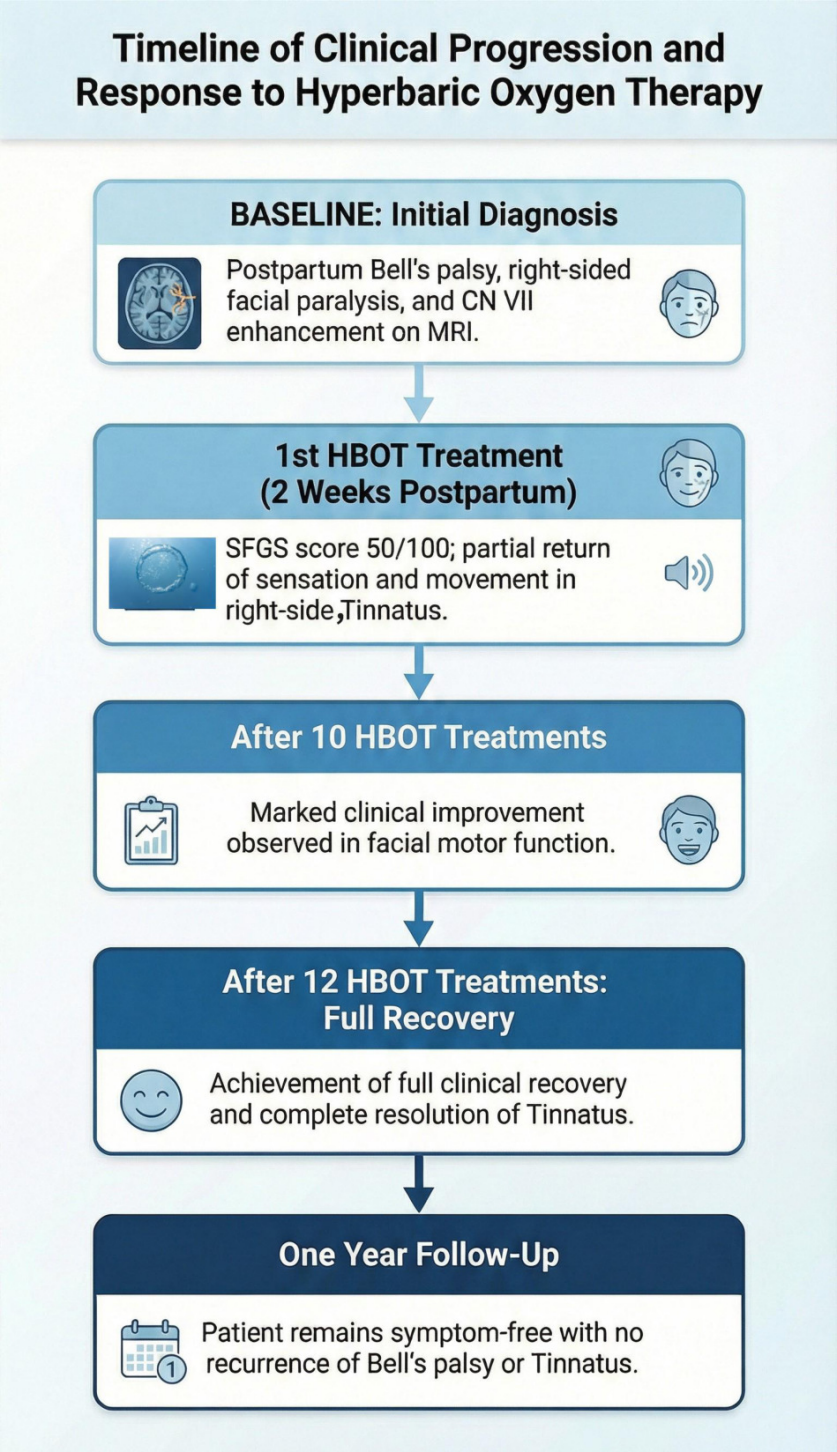
Timeline of clinical progression and response to hyperbaric oxygen therapy.

Two weeks postpartum, with no improvement after daily 60 mg prednisone (Figure 1A), she was referred for HBOT with no other treatment besides the Prednisone. She was prescribed to receive 20 sessions, 5 days per week, each involving 90 min of 100% oxygen at 2 absolute atmospheres with 5-minute air breaks every 20 min. Facial function was assessed using the Sunnybrook Facial Grading System (SFGS) (19) (Supplement) by a physiotherapist, with video documentation every two sessions. Notably, after the first session, she reported a partial return of sensation and movement on the right side of her face, in addition to progressive improvement of the tinnitus in the right ear, with continued improvement after each session. By the tenth session, her SFGS score had improved from 33% to 83% (Figure 1B), after 12 sessions, she achieved full recovery, and no longer needed an eyelid weight for eye closure (Table 1). In addition, the patient reported no tinnitus. One-year follow-up demonstrated no recurrence of symptoms (see Figure 2 for timeline details). Notably, the patient's previously positive anti-dsDNA antibodies turned negative in two consecutive tests following HBOT.

**Table 1:** Comparison of facial function scores using the Sunnybrook facial grading system (SFGS).

Parameter	Pre HBOT treatment	10 Session HBOT treatment	12 Session HBOT treatment
Resting symmetry			
Eye	1	0	0
Cheek	1	0	0
Mouth	1	0	0
Resting symmetry score	3/5 moderate asymmetry	0/5 normal symmetry	0/5 normal symmetry
Voluntary movement			
Forehead wrinkle	2	4	5
Gentle eye closure	3.5	5	5
Open mouth smile	3.5	4	5
Snarl	2	3.5	5
Lip pucker	1	4	5
Vol movement total	12	20.5	25
Vol movement score (Total×4)	**48/100**	**82/100**	**100/100**
Synkinesis			
Synkinesis score	NR	NR	NR
Composite score	**33**	**82**	**100**

The bold values are the sum of the subsections and the composite score.

## Discussion

**Figure 3:**
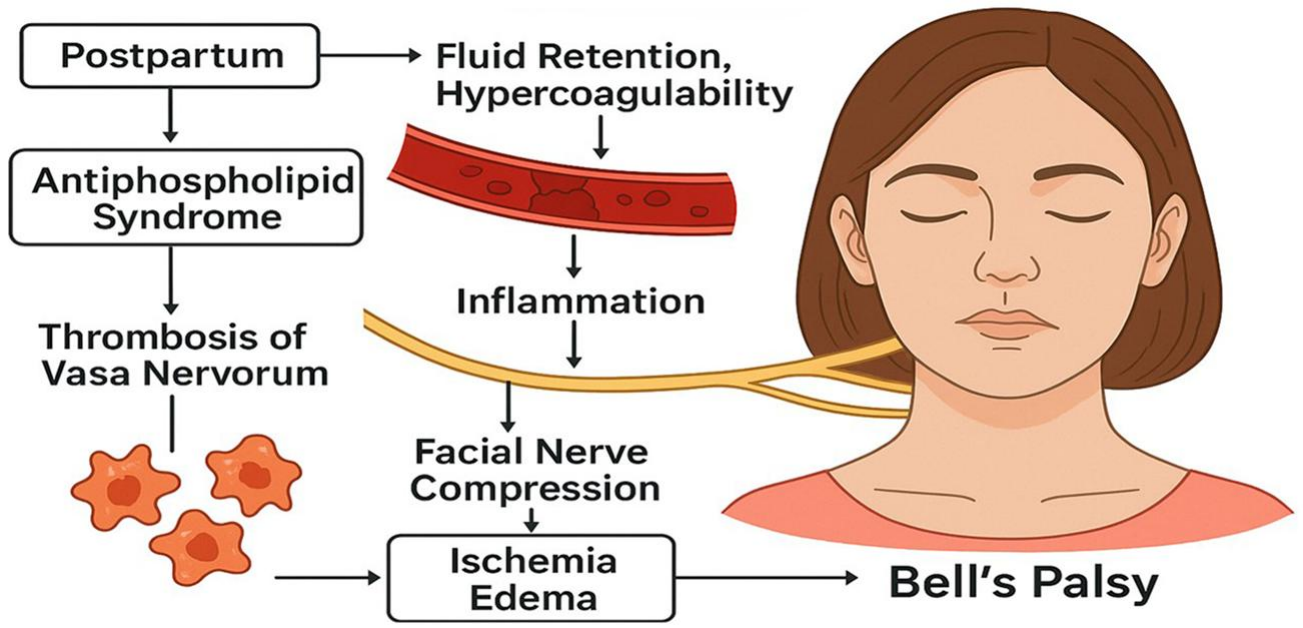
The pathophysiological mechanism of post-partum Bells palsy.

This case illustrates postpartum Bell's palsy in the context of APS, likely driven by pregnancy-related physiological changes, inflammation, and immune-mediated microvascular ischemia (Figure 3 and Table 2). The patient's lack of response to corticosteroids but rapid improvement with HBOT suggests that ischemia and hypoxic injury, rather than demyelination alone, may have played a primary role in her facial nerve dysfunction.

**Table 2:** The pathophysiological cascade that likely contributed to the development of bell's palsy in the present patient's setting.

Factor	Mechanism	Contribution to this case
Pregnancy-related changes	Fluid retention, hormonal shifts, hypercoagulability	Perineural edema and vascular stasis
Inflammatory responses	Pregnancy and postpartum-associated immune modulation	Increased vascular permeability, local inflammation and edema
Underlying APS	Autoimmune-mediated endothelial dysfunction, thrombosis of vasa nervorum	Elevated risk of microvascular ischemia of the facial nerve
Facial nerve ischemia	Microvascular occlusion and endothelial dysfunction of facial nerve supply	Reduced perfusion and local tissue hypoxia
Nerve compression within canal	Edema-induced compression in the bony facial canal	Mechanical compression of the facial nerve
Impaired nerve conduction	Ischemia and edema disrupt axonal function	Acute-onset unilateral facial paralysis (Bell's palsy)
Failure of corticosteroid monotherapy	Limited efficacy against combined ischemic and ischemia induce pathology	No improvement on prednisone alone
HBOT intervention	Enhanced oxygen delivery, reduced edema, improved microvascular perfusion	Rapid symptom improvement and full recovery within 12 sessions

HBOT addresses these components by significantly increasing plasma oxygen partial pressure, enhancing oxygen delivery to ischemic nerve tissue, and reducing inflammatory edema that compresses the facial nerve (20). Moreover, HBOT has been shown to induce mitochondrial biogenesis and activate SIRT1, promoting neuronal survival and repair (21, 22).

The beneficial effects of HBOT observed in this patient align with cumulative data on the use of HBOT in peripheral nerve injuries. A comprehensive systematic review by Brenna et al. from the University of Toronto meticulously summarized the available data, concluding that HBOT was beneficial in the majority of six human trials, with positive outcomes reported in 83% of cases (23). Subsequent studies published after the Brenna et al. review have further illuminated HBOT's potential, demonstrating enhanced motor function, reduction in neuroinflammation, improved mitochondrial function, and prevention of neuronal apoptosis (24, 25). These findings support the mechanistic rationale for using HBOT in cases of facial nerve ischemia and compression. Intriguingly, the disappearance of anti-dsDNA antibodies after HBOT suggests potential immunomodulatory effects, consistent with previous studies in murine lupus models (26).

The strength of this case report lies in the comprehensive documentation of symptom progression and resolution, offering valuable insights for clinical practice. However, the limitations include the single-case design, which restricts generalizability and preclude definitive conclusions regarding sustained efficacy. From a research perspective, further controlled studies are needed to establish standardized HBOT protocols and clarify its role in managing Bell's palsy in high-risk populations.

## Conclusion

This case underscores the contribution of pregnancy-related physiological changes, inflammation, and vascular compromise to postpartum Bell's palsy in the setting of APS. HBOT's combined effects on oxygenation, inflammation control, and vascular repair likely underpinned the rapid, complete resolution of symptoms in this patient. While additional evidence is required, this case suggests HBOT is a promising adjunct in the postpartum setting.

## Data Availability

The original contributions presented in the study are included in the article/Supplementary Material, further inquiries can be directed to the corresponding authors.
